# Ecdysone-Related Biomarkers of Toxicity in the Model Organism *Chironomus riparius*: Stage and Sex-Dependent Variations in Gene Expression Profiles

**DOI:** 10.1371/journal.pone.0140239

**Published:** 2015-10-08

**Authors:** Rosario Planelló, Óscar Herrero, Pablo Gómez-Sande, Irene Ozáez, Fernando Cobo, María J. Servia

**Affiliations:** 1 Grupo de Biología y Toxicología Ambiental, Facultad de Ciencias, Universidad Nacional de Educación a Distancia, UNED, Paseo de la Senda del Rey 9, 28040 Madrid, Spain; 2 Departamento de Zoología y Antropología Física, Universidad de Santiago de Compostela, USC, Campus Sur s/n, 15782 Santiago de Compostela, Spain; 3 Estación de Hidrobioloxía “Encoro do Con”, EHEC, Universidad de Santiago de Compostela, USC, Castroagudín s/n, 36617 Vilagarcía de Arousa, Pontevedra, Spain; 4 Departamento de Biología Animal, Biología Vegetal y Ecología, Facultad de Ciencias, Universidade da Coruña, UDC, Campus da Zapateira s/n, 15008 A Coruña, Spain; Laboratoire Arago, FRANCE

## Abstract

Despite being considered a model organism in toxicity studies, particularly in assessing the environmental impact of endocrine disrupting compounds (EDCs) and other chemicals, the molecular basis of development is largely unknown in *Chironomus riparius*. We have characterized the expression patterns of important genes involved in the ecdysone pathway from embryos to pupa, but specially during the different phases of *C*. *riparius* fourth larval instar, according to the development of genital and thoracic imaginal discs. Real-Time PCR was used to analyze: *EcR* and *usp*, two genes encoding the two dimerizing partners of the functional ecdysone receptor; *E74*, an early response gene induced by ecdysteroids; *vg* (vitellogenin), an effector gene; *hsp70* and *hsc70*, two heat-shock genes involved in the correct folding of the ecdysone receptor; and *rpL13*, as a part of the ribosomal machinery. Our results show for the first time stage and sex-dependent variations in ecdysone-responsive genes, specially during the late larval stage of *C*. *riparius*. The induction in the expression of *EcR* and *usp* during the VII-VIII phase of the fourth instar is concomitant with a coordinated response in the activity of the other genes analyzed, suggesting the moment where larvae prepare for pupation. This work is particularly relevant given that most of the analyzed genes have been proposed previously in this species as sensitive biomarkers for the toxicological evaluation of aquatic ecosystems. Identifying the natural regulation of these molecular endpoints throughout the *Chironomus* development will contribute to a more in-depth and accurate evaluation of the disrupting effects of EDCs in ecotoxicological studies.

## Introduction

Metamorphosis, the transition from the larval stage to the adult, involves important molecular and cellular alterations and results in dramatic morphological and physiological changes in holometabolous insects. These variations demand the loss of embryo tissues and the differentiation or the *novo* formation of other ones. The harmonized action of two hormones, 20-hydroxyecdysone (20E) and juvenile hormone (JH), is responsible for coordinating insect growth and development, and the balance between them defines the outcome of each developmental transition. In a larva-to-larva molt a JH titer is needed, while metamorphosis takes place when the JH level drops and a 20E titer occurs during the final larval instar [1].

The wide variety of insect groups within Holometabola limits the ability to generalize about development control. The effects of hormones on a group may not be the same in others because of different growth patterns and cell specificities. These differences in responses to hormones add complexity to the interpretation of many findings, and generalizing about common mechanisms in evolutionarily distant and well-described groups should be done cautiously.

The steroid signaling mechanisms have been extensively studied in *Drosophila melanogaster* and *Manduca sexta*, model insects in developmental studies and neurobiology, and more recently in *Tribolium castaneum* and *Aedes aegypti*, an important pest and vector for several diseases, respectively [2]. In contrast, there is little information on the stage-specific expression of hormonal-related genes during the developmental process in the midge *Chironomus riparius*, which is considered by US EPA and OECD a model organism for ecotoxicity testing and has several internationally validated guidelines [3–5] used for regulatory purposes in environmental toxicology. This species has also been selected as a reference organism in the study of the potential adverse effects of endocrine disrupting chemicals [6] and as a model to evaluate endocrine disrupting effects in aquatic invertebrates for the European IDEA project [7]. Although some changes could somehow be explained in *Chironomus* from known patterns of closely related species, such as *Drosophila* or *Aedes*, physiological differences regarding their life cycle or feeding behavior make interesting a more in-depth study of *Chironomus* development.

Transcriptional response to ecdysteroids in insects requires the action of two nuclear receptor superfamily members, the ecdysone receptor (EcR) and the ultraspiracle (USP) [8]. The activation of the EcR/USP heterodimer initiates the cascade expression of ecdysone-responsive genes that leads to drastic changes in cell proliferation, apoptosis and the disappearance of larval organs, as well as the differentiation of adult tissues.

The expression of *EcR* and *usp* has been well described during *D*. *melanogaster* development, from embryos to adults [9,10]. Different isoforms of these genes have been also characterized in many insects other than *Drosophila* and dissimilar time-space expression profiles have been reported ([11] and references therein). For example, multiple forms of *EcR* and *usp* and their complex regulation have been observed in *M*. *sexta* [12], and cDNAs from different isoforms of both genes have been characterized in *Chironomus tentans* [13,14]. Moreover, the ecdysone receptor gene has been described recently in *C*. *riparius* [15], although little is known about the developmental expression profile of *EcR* and *usp* in this species, specially during the fourth larval instar, which is the most critical stage for individuals undergoing metamorphosis.

Deep into the ecdysone-responsive genes cascade, *E74* is one of the early genes induced by ecdysteroids. It has been described as a transcription factor that plays a critical role at the time of metamorphosis in *D*. *melanogaster* [16,17] and *M*. *sexta* [18], where expression patterns of this gene correlate with pupal commitments.

As a part of their development, insects supply their eggs with protein, lipids, carbohydrates and other resources for feeding the growing embryos. Several types of YPP (Yolk Protein Precursor) are accumulated by insect oocytes in response to endogenous estrogens, but vitellogenin (Vg) is the most abundant. Multiple *vg* genes/cDNAs have been sequenced in many insects [19]. The synthesis of Vg is regulated at transcriptional level and *vg* gene is normally silenced in males and immature females probably due to low levels of estrogens in plasma, although may be activated by (xeno-)estrogens. Therefore, Vg has been proposed as a useful biomarker in the evaluation of the estrogenic effects of pollutants in vertebrates and invertebrates, including *C*. *riparius* [20,21]. Nevertheless, there is a lack of information about expression patterns throughout the development of this midge.

It is worth highlighting the importance of heat-shock proteins (HSPs) in the folding and maturation of steroid hormone receptors. Among all HSPs, the HSP70 family represents one of the most highly conserved proteins identified to date in all organisms in which they have been described. The family includes constitutive members such as cognate proteins (HSC70), highly abundant in normal cellular conditions, and inducible proteins (HSP70) under a broad spectrum of physical and chemical stress conditions [22]. A functional relationship between steroid hormones and HSPs has been reported and it is known that HSP70, HSC70 and HP90 play an important role in the folding and maturation of steroid hormone receptors and different transcription factors [23]. There have been recent advances in the molecular description of *hsp70* genes in a variety of insects, including *C*. *riparius*, as well as in their evaluation in response to different environmental stressful conditions [24–27]. It also has been suggested that *hsc70*/*hsp70* ratio may be a potential indicator of polluted environments [28]. On the contrary, the information about the response of these genes across developmental stages in *C*. *riparius* in relation to their chaperone activity and their role in the folding of the ecdysone receptor is still scarce.

Finally, ribosomal protein genes are essential for cellular growth and development, since they code for the necessary machinery for protein synthesis, together with the four rRNAs (28S, 18S, 5.8S, 5S). They are considered housekeeping genes and they are constitutively expressed. Furthermore, additional ribosomal functions have been described for some ribosomal proteins, included L13, such as the control of transcriptional regulation, specially in the development and metamorphosis of insects. To date, more than 80 different types of ribosomal proteins have been identified in eukaryotes, but only the genes encoding for six ribosomal proteins have been characterized in *C*. *riparius*. Despite their constitutive expression, alterations in the levels of some ribosomal proteins under exposures to different xenobiotics have been reported in this species [29–32]. Due to the recent description of ribosomal genes as potential biomarkers of toxicity and given the lack of information on their potential role during development, evaluating possible ontogenetic-dependent changes is of particular interest.

In the present work we characterize the expression patterns of important genes in the ecdysone pathway in *C*. *riparius*: *EcR*, *usp*, *E74*, *vg*, *hsp70*, *hsc70* as well as the housekeeping gene *L13*. They have been analyzed from embryo to pupa, but specially throughout the fourth larval instar, which is usually the selected stage for ecotoxicity testing. We report for the first time in this organism a developmental stage and sex-dependent expression of all the analyzed genes and describe the coordinated response that triggers the mechanisms to start the metamorphosis.

## Material and Methods

### Ethics statement

Sampling and protocols used in this study conform to the administrative and ethical laws of the regional government (Xunta de Galicia) and did not involve endangered or protected species.

### Test animals and culture conditions

The experimental animals were the aquatic embryos, larvae and pupae of the midge *Chironomus riparius*. They were reared in the laboratory in strict accordance with the recommendations given in standardized international guidelines [3–5]. They were grown from egg masses in aqueous culture medium (0.5 mM CaCl_2_, 1 mM NaCl, 1 mM MgSO_4_, 0.1 mM NaHCO_3_, 0.025 mM KH_2_PO_4_, 0.01 mM FeCl_3_) supplemented with nettle leaves, commercial fish food, and cellulose tissue in polyethylene tanks (500 ml). Cultures were maintained under constant aeration at 20°C and standard light-dark periods 16:8. Additionally, a brief approach was conducted using larvae from natural populations of the midge collected in the Sar river (Galicia, NW Spain).

**Fig 1:**
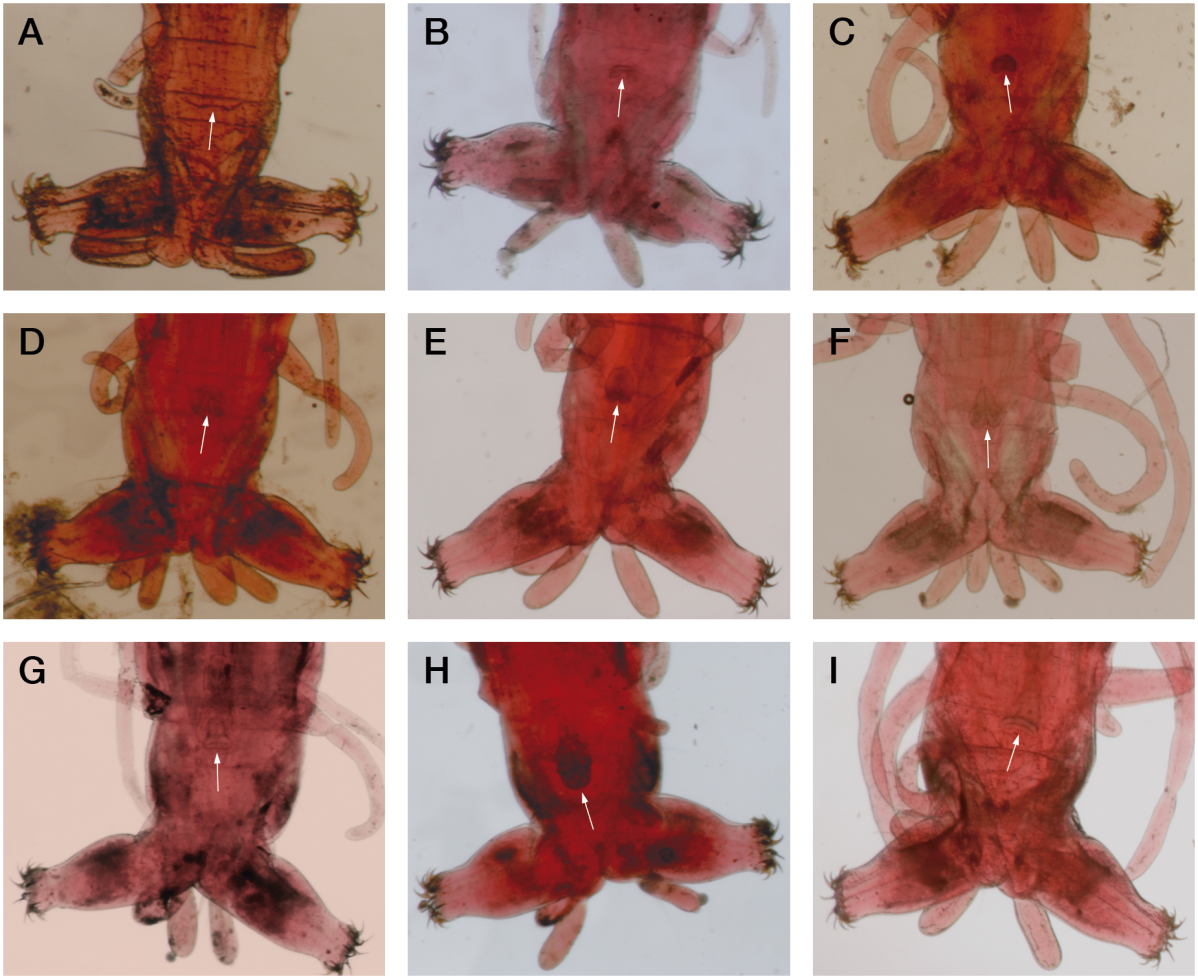
Representative images of the separation of larvae carried out in this work. Fourth instar larva were aged and sexed based on the development of genital and thoracic imaginal discs, according to [33], who divided the fourth instar into nine phases (phase I to phase IX). In order to reduce stressful conditions to larvae, phases were grouped into five broader categories (I-II; III-IV; V-VI; VII-VIII and IX, respectively). A. (I-II); B. (III-male); C. (IV-female); D. (V-male); E. (VI-female); F. (VII-male); G. (VIII-female); H. (IX-male) and I. (IX-female).

Embryos were selected 48 h after ovoposition. First to fourth instar larvae were identified by measuring head capsule width, while the phases within the fourth instar were established from the development of genital and thoracic imaginal discs according to [33], where the 4^th^ instar was divided into nine phases (phase I to phase IX). For the sake of promptness, and in order to reduce stressful conditions to larvae, fourth instar individuals were grouped into five broader categories that could be easily established under the microscope: 1) I-II phase; 2) III-IV phase; 3) V-VI phase; 4) VII-VIII phase; and 5) IX phase. Additionally, the sex of the larvae was established where possible. Larval individuals were sexed and aged under a binocular microscope (x40 magnification) (Fig 1), and four different samples of each category were prepared for analysis of expression profile during embryo stage, larval development and pupation.

### RNA extraction

RNA was extracted from a total of 4 egg masses and 20 frozen individuals (larvae or pupa, depending on the developmental stage or phase), divided into groups of five, using a guanidine isothiocyanate based method, performed with a commercial kit (TRIzol, Invitrogen). To isolate embryo RNA, a prior treatment was performed to remove the gelatinous cover of the egg mass. The egg mass was treated with 1xPBS (137 mM ClNa, 2.7 mM KCl, 8.1 mM Na_2_HPO_4_, 1.5 mM KH_2_PO_4_) and 0.2% sodium hypochlorite until the gelatinous cover disappeared. Subsequently, the eggs were washed several times with 1xPBS until the sodium hypochlorite was completely eliminated. Briefly, eggs or frozen material were homogenated in one volume of TRIzol and left for 5 min at room temperature. Then, 0.2 volumes of chloroform were added to each sample, mixed and left for 5 min at room temperature. Subsequently, the samples were centrifuged for 15 min at 4°C and 15000 g. Following transfer of the aqueous phase, the RNA was finally recovered by isopropyl alcohol precipitation (0.5 v/v), washed with 70% ethanol, and resuspended in DEPC water. The RNA was then treated with RNase-free DNase (Roche) followed by phenolization. The quality and quantity of total RNA were determined by agarose electrophoresis and absorbance spectrophotometry (Nanodrop1000, Thermo), and the purified RNA was finally stored at -80°C. RNA samples were sent to the Biology and Environmental Toxicology Group (UNED), where expression assays were carried out.

#### Reverse transcription and Real-Time PCR

After checking RNA integrity in 1.5% agarose gels, reverse transcription was performed with 0.5 μg of the isolated RNA, and 0.5 μg oligo dT_20_ primer (Sigma) was used with M-MLV enzyme (Invitrogen). The cDNA obtained was used as template for the Polymerase Chain Reaction (PCR).

Quantitative Real-Time PCR (qRT-PCR) was used to evaluate the mRNA expression profile of *EcR*, *usp*, *E74*, *vg*, *hsp70*, *hsc70* and *L13* genes during different developmental stages (embryo, 1^st^, 2^nd^, 3^rd^ and 4^th^ instar larvae, and pupa). The q-PCR was performed using CFX96 thermocycler (BioRad) and SsoFast EvaGreen Supermix (BioRad), with 25 ng of cDNA and 300nM of forward and reverse primers per reaction. Ribosomal gene *26S*, *actin* and *GAPDH* were employed as endogenous reference genes [34,35]. *EcR*, *usp*, *E74*, *hsc70* and *hsp70* primers are described in [36,37]. The *vg* primers were designed from *C*. *riparius* sequences present in the database: # HQ260608 [38]. The *L13* primers are described in [39].

To accurately determine the efficiencies of the PCR reactions, reaction mixtures with template dilutions 1:2 in five steps were also run in the same PCR conditions, and the slopes of the regression curves were calculated (R^2^>0.98). The Real-Time PCR primers sequences used in this study, efficiencies and fragment size of each gene-specific pair of primers are listed in Table 1. The acceptable range for PCR efficiencies calculated using standard curve serial dilution experiments is 90–110% [40].

**Table 1:** Primers used for Real-Time PCR amplification of the genes studied in *C*. *riparius*.

Gene	Name	DNA sequence (5′-3′)	Fragment size (bp)	Efficiency (%)
*actin*	Forward	GATGAAGATCCTCACCGAACG	201	104
	Reverse	CGGAAACGTTCATTACCG		
*GAPDH*	Forward	GGTATTTCATTGAATGATCACTTTG	110	96.6
	Reverse	TAATCCTTGGATTGCATGTACTTG		
*26S*	Forward	TTCGCGACCTCAACTCATGT	220	90.4
	Reverse	CCGCATTCAAGCTGGACTTA		
*EcR*	Forward	CCATCGTCATCTTCTCAG	180	106.6
	Reverse	TGCCCATTGTTCGTAG		
*usp*	Forward	GCCCAATCATCCGTTAAGTGG	114	108.1
	Reverse	CGTTTGAAGAATCCTTTACATCC		
*E74*	Forward	TCTTACTGAAACTTCTTCAAGATCG	111	103.2
	Reverse	GCTTTGAGACAGCTTTGGAATCG		
*vg*	Forward	GATTGTTCCATGTGCAG	215	112.3
	Reverse	TTTGAGTATGGTGGAGAATC		
*hsp70*	Forward	ACTTGAACCAGTTGAGCGT	132	103.8
	Reverse	TTGCCACAGAAGAAATCTTG		
*hsc70*	Forward	CGTGCTATGACTAAGGACAA	239	99.3
	Reverse	GCTTCATTGACCATACGTTC		
*rpL13*	Forward	AAGCTGCTTTCCCAAGAC	351	109.3
	Reverse	TTGGCATAATTGGTCCAG		

Real-Time PCR was run in the following cycling conditions: initial denaturation at 95°C for 3 min, 35 cycles of 95°C denaturation for 5 s, 58°C annealing for 15 s and 65°C elongation for 10 s. To verify the accuracy of each amplicon, a melting curve analysis was carried out after amplification BioRad CFX Manager 2.1 software was used to calculate the mRNA levels by the normalized gene expression (2^-ΔΔCT^) against three endogenous reference genes (26S, *actin* and *GAPDH*). Additionally, several statistical analyses were carried out to deeply interpret the results. A total of four egg masses and 20 individuals of each developmental category (1^st^, 2^nd^ and 3^rd^ instar larvae, five phases of the 4^th^ instar, and pupa, respectively) were used in this study. For each category, samples were divided into four groups containing one egg mass, five larvae or five pupae, as appropriate. To avoid variations caused by experimental procedures each group was analyzed three times (three PCR amplification replicates) and each q-PCR replicate was run in duplicate wells.

### Determination of ecdysteroids levels during development

Ecdysteroids levels were measured via competitive Enzyme Immunoassay (EIA) 20-Hydroxyecdysone EIA kit (A05120) SPI-Bio kit (Bertin Pharma), using 20E and 20E acetylcholinesterase as the standard and enzymatic tracer, respectively. For sample preparation, individual larvae were weighed and homogenized in 250 μl of iced-cold 75% aqueous methanol and centrifuged at 13000 g at 4°C for 15 min. Precipitates were resuspended in an additional 100 μl of aqueous methanol and kept on ice for 30 min. After a new centrifugation as above, the supernatant was combined with the previous one. Samples were vacuum dried and resuspended in 100ul of enzyme immunoassay (EIA) buffer. Ellmann reagent was used for the chromogenic reaction and absorbance was read at 415 nm. All assays were performed in triplicate.

### Statistical analysis

Normality and homoscedasticity of data were tested using the Shapiro-Wilk and Levene tests respectively. Datasets were, if necessary, normalized using natural log (ln) or square root transformations. The levels of the specific gene transcripts were analyzed with ANOVA, followed by Games Howell’s or Tukey’s post Hoc tests when appropriate. If transformed data were not homogeneous or normally distributed the Kruskal-Wallis test was used, and the differences between pairs were analyzed using the multiple comparisons of mean ranks for all groups test of [41]. Differences were considered significant at p<0.05.

A cluster analysis method for grouping the different developmental stages was applied using the mean expression values of each gene. All the analyses were performed using SPSS 21 (IBM).

## Results

### Expression patterns of hormonal receptors and ecdysone-inducible genes

At the molecular level, the first step in the action of 20E consists in its binding to a complex of two nuclear receptors: the ecdysone receptor (EcR) and its heterodimerization partner ultraspiracle (USP). The induction of *EcR* by the 20E controls the transcription of a set of early response genes, as *E74* among others.

**Fig 2:**
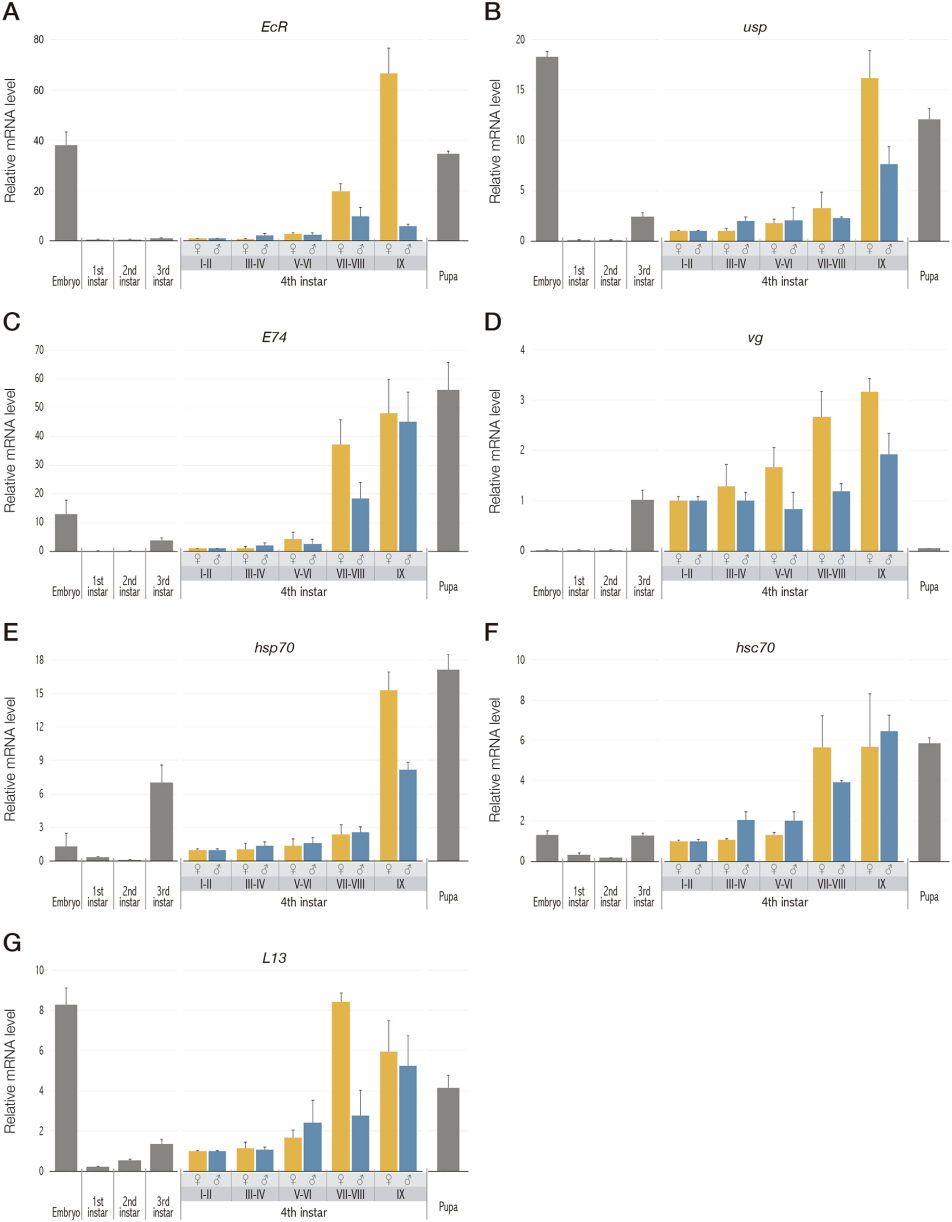
Ontogenetic variations in the expression pattern of the genes analyzed. Transcriptional levels of genes from *C*. *riparius* involved in the ecdysone-related pathway (*EcR*, *usp*, *E74* and *vg*), the folding and maturation of steroid hormone receptors (*hsp70* and *hsc70) and* the synthesis of the ribosomal protein L13. Gene expression was measured from embryo to pupa stages of development. The mRNA values were calculated relative to *actin*, *GAPDH* and *26s* as reference genes. Each bar is the mean ± SE obtained from four independent samples, each with three experimental replicates. A total of 4 egg masses and 20 larvae of each stage or phase were used. All the analyzed genes showed significant differences among the different stages (Fig 3).

**Fig 3:**
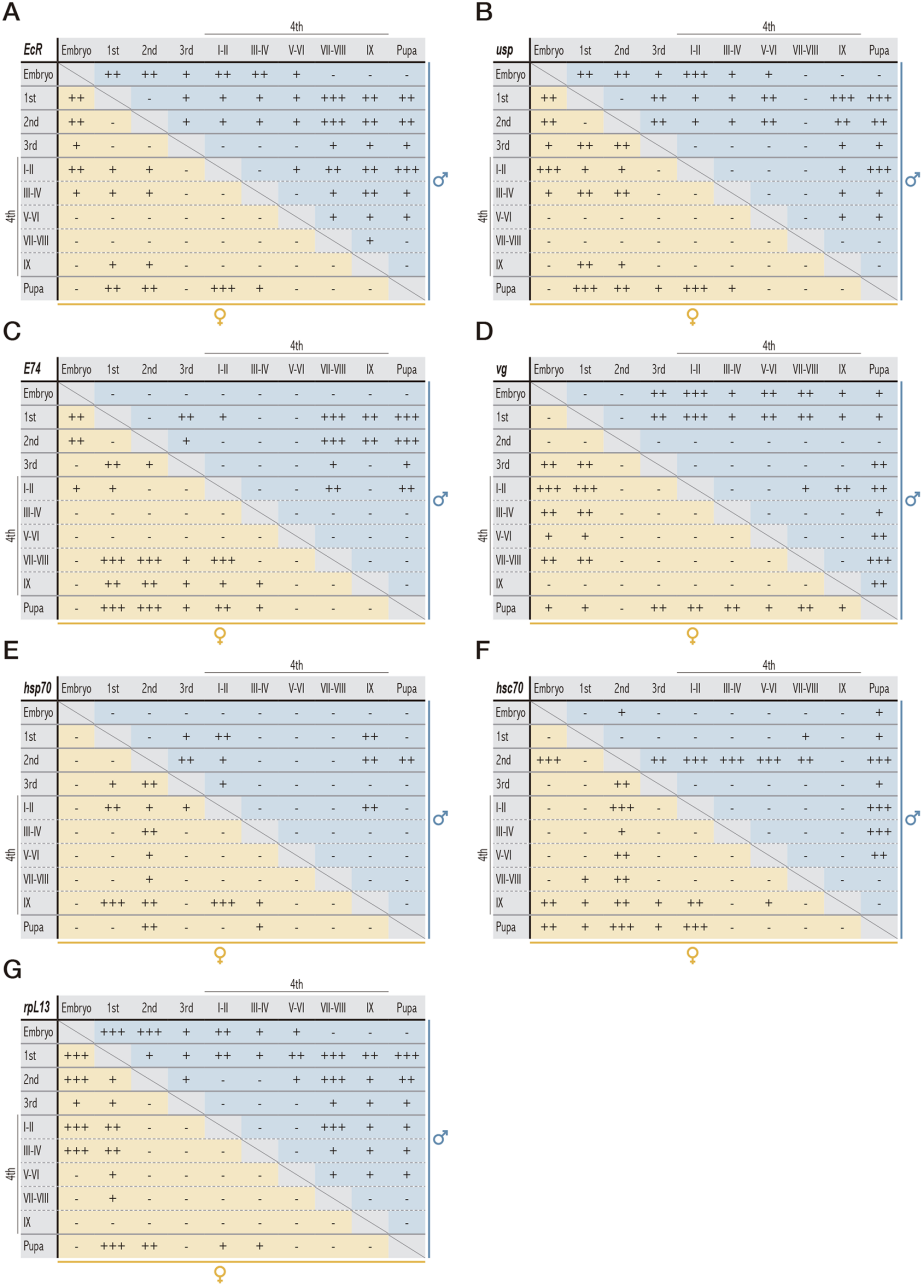
Significant differences in the expression pattern of the genes analyzed. All the analyzed genes showed significant differences among the different stages according to Kruskal-Wallis Post hoc test. Three p values were tested in all cases.—(no differences), **+** (p < 0.05), **++** (p < 0.01), **+++** (p < 0.001).

Our results show a coordinated expression pattern with a stage dependent variation of all these genes, especially when comparing early larval stages with late larval stages or even pupae. We found similar responses for *EcR*, *usp* and *E74* genes, which consisted of a high transcriptional activity in the embryo stage that dropped drastically at the 1^st^ larval instar. However, it is noteworthy that although transcript levels remained low throughout early phases of the 4^th^ instar, a strong induction was clearly measured for late phases and pupa stages. As shown in Figs 2A and 3A, *EcR* was strongly and significantly induced at VII-VIII phase and reached maximum levels in the IX phase (prepupa) (respectively, 60-fold and up to 260-fold when compared to the lowest level measured in this study). Interestingly, *usp* showed a similar trend but with lower transcriptional rates than those of *EcR*, with maximal inductions of 30-fold and 130-fold respectively in VII-VIII and IX phases (Figs 2B and 3B). Apart from these developmental changes, the expression pattern of both *EcR* and *usp* showed a sex-dependent response during the 4^th^ larval stage (Fig 2A and 2B), with significant differences between males and females, particularly in the onset of metamorphosis (VII-VIII and IX phases).

The expression pattern of *E74* (Figs 2C and 3C) showed an induction in late larvae (VII-VIII and IX phases) in unison with maximal values of *EcR*, and reached its highest values in pupation (50-fold when compared to the lowest level measured in this study) concomitantly with a slight drop in the expression level of the ecdysone receptor gene. This result suggests that this up-regulation might be part of the early gene response to ecdysteroids that occurs specifically at the point of pupa commitment. Furthermore, as an example of effector gene in the ecdysone pathway, we focused on the study of *vg*, which is considered as a reproductive biomarker. Interestingly, *vg* gene showed a tendency to increase throughout the fourth stage concomitantly with the induction detected in *EcR* levels, and followed by a sharp and significant decline in pupal stage (Figs 2D and 3D).

Finally, a brief study focused in late development (from third instar larvae to pupa) was carried out using larvae from natural populations of the midge. Similar transcriptional response of these genes was observed when compared to laboratory cultures, with low levels detected in early developmental stages and strong inductions at late larval and pupal stages (S1A–S1D Fig).

### Changes in the expression of the 70-kDa heat-shock inducible and cognate genes

Considering the role of the HSP70 and HSC70 as chaperones involved in EcR folding, the basal expression of *hsp70* and *hsc70* genes was evaluated throughout the *C*. *riparius* development, with special interest in the different phases described during the fourth instar larval stage. Both genes showed significant differences among the different stages (Fig 3E and 3F). Similarly to the expression profiles observed for ecdysone-responsive genes, notable differences between the early and late stages were observed for heat-shock genes. Thus, *hsp70* transcript levels were significantly high at IX phase (prepupa) and remained high in the pupal stage (15-fold when compared to the lowest level measured in this study), while a clear increase was also detected for 3^rd^ instar larvae (Fig 2E). Similarly, relative low levels of *hsc70* mRNA were found at early development and a strong and significant activation was detected in late larval stages (VII-VIII and IX phases) and continued in pupae (about 6-fold more than in earlier phases) (Fig 2F).

It is worth pointing out that the responses of both *hsp70* and *EcR* genes occurred concomitantly, with low levels at early 4^th^ instar larvae and a maximum transcriptional activity at late 4^th^ instar larval phases and pupae.

The study of larvae in natural populations revealed a similar response of these genes throughout the fourth larval instar, compared with laboratory cultures (S1E and S1F Fig).

### Variations in the expression levels of the ribosomal housekeeping gene *L13*

Real-Time PCR experiments were carried out to evaluate the expression patterns of *L13* gene, and changes in *L13* transcript levels were detected throughout the different stages analyzed. Fig 2G shows a coordinated response of *L13* and the remaining studied genes, which were strongly upregulated at the end of larval development (from VII-VIII phase onwards). Deep into the 4^th^ larval stage, up to an 8-fold induction was observed when compared to early fourth instar phases. It is also remarkable the high expression level of this gene at embryo stage.

Similarly to the expression profiles observed in larvae from laboratory, notable differences between early and late stages were observed in natural populations (S1G Fig).

### Analysis of sex-dependent variations

Taking into account the role of ecdysone-responsive genes in sexual differentiation and also in the production of energy reserves in mature eggs that will be deposited by adult females, we consider that sex-dependent changes could determine transcriptional processes. As a first approach to confirm this assumption, data of 4^th^ instar larva were preliminarily analyzed taking into account that, except for the I-II phase, samples of each group into the 4^th^ instar were separated by sex when collected.

Apart from the developmental changes described previously, the expression pattern of both *EcR* and *usp* showed a sex-dependent response during the 4^th^ larval stage (Fig 2A and 2B), with significant differences between males and females, particularly in the onset of metamorphosis (VII-VIII and IX phases). Although differences between the expression levels of males and females were also detected for *E74* and *vg* (Fig 2C and 2D), these variations were not as remarkable as those found in the case of EcR and usp. Furthermore, except for the prepupa values of *hsp70*, heat-shock genes behaved similarly in males and females (Fig 2E and 2F). Finally, the transcriptional response of *L13* was quite similar between sexes, except for a higher response of females from phase VII-VIII.

Results obtained in larvae from field populations also showed a sex-dependent variation in the transcriptional response of the analyzed genes, with a transcriptional tendency similar to that described for laboratory cultures (S1 Fig).

### Analysis of the expression profile dataset

**Fig 4:**
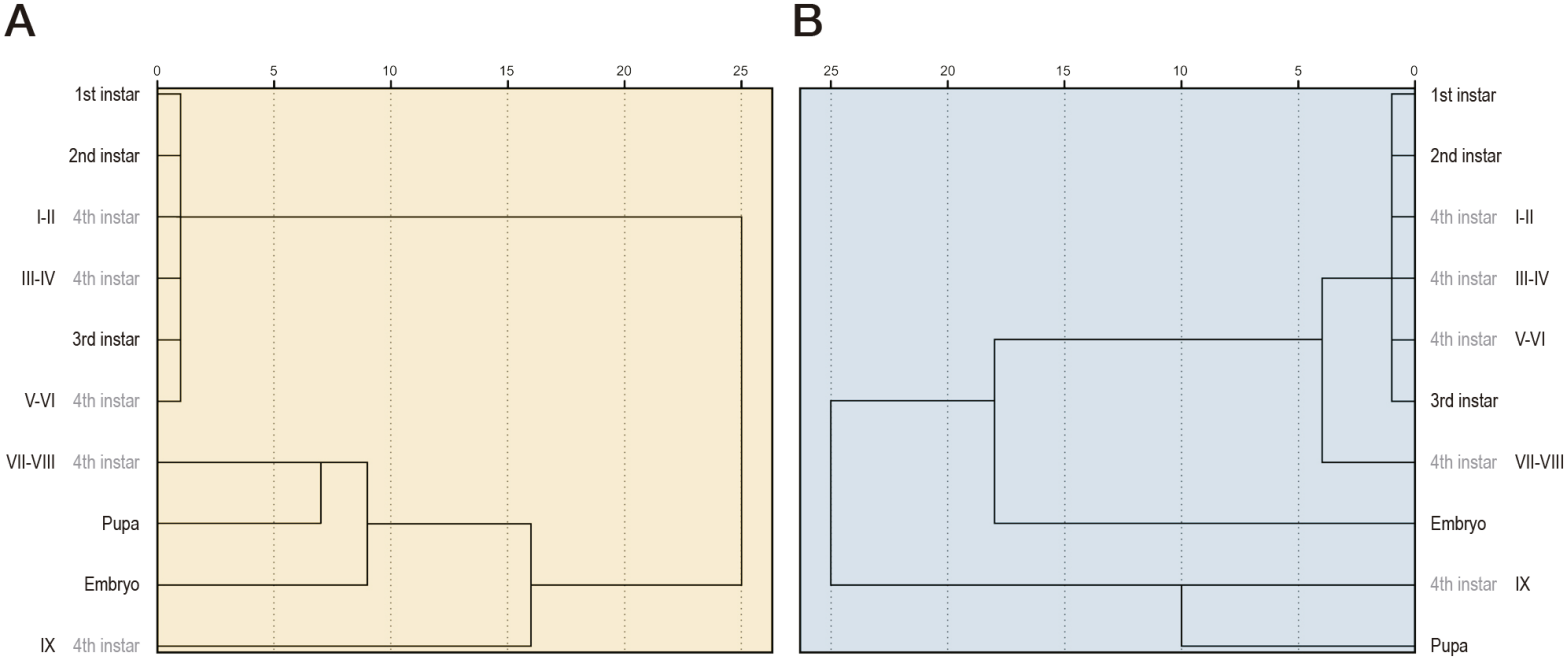
Hierarchical clustering dendrogram of the groups established for the expression analysis of ecdysone related genes. Cluster analysis arranges biological samples into groups based on the expression levels measured in female (A) and male (B) larvae. Relationships among samples are represented by a dendrogram whose branch lengths reflect the degree of similarity between them as assessed by pairwise comparisons of gene expression profiles. Two groups of developmental stages or phases are clearly separated with slight differences between sexes: 1) 1^st^, 2^nd^, 3^rd^ instar larvae and early 4^th^ instar phases; and 2) late 4^th^ instar larvae, pupa and embryo. The root represents the whole data set and a leaf corresponds to a single object in it. An internal node represents the union of all objects in its sub-tree. The weight of an internal node represents the distance between its two child nodes.

Taking into account the mean value for each gene in each larval stage/phase, an analysis of the entire dataset based on the sex of the samples was finally carried out. Clustering is an exploratory tool for looking at associations within gene expression data that allowed us to hypothesize about relationships between gene response and class/group of larvae. According to the response of the analyzed genes, the hierarchical clustering dendrograms obtained (Fig 4) found two main groups of biological samples in our data: 1) a group containing 1^st^, 2^nd^, 3^rd^ instar larvae and early 4^th^ instar phases; and 2) a group including the late phases of the 4^th^ stage (VII-VIII and IX) and the pupa, as well as the embryo, which showed extreme values depending on the gene.

In the case of larvae from natural populations, the cluster analysis based on the expression levels found two groups of biological samples in our data. On the one hand, the 3^rd^ instar larvae and early 4^th^ instar phases; and on the other hand, the late phases of the 4^th^ stage and pupa (S2 Fig).

#### Variations in ecdysteroid levels during larval development

**Fig 5:**
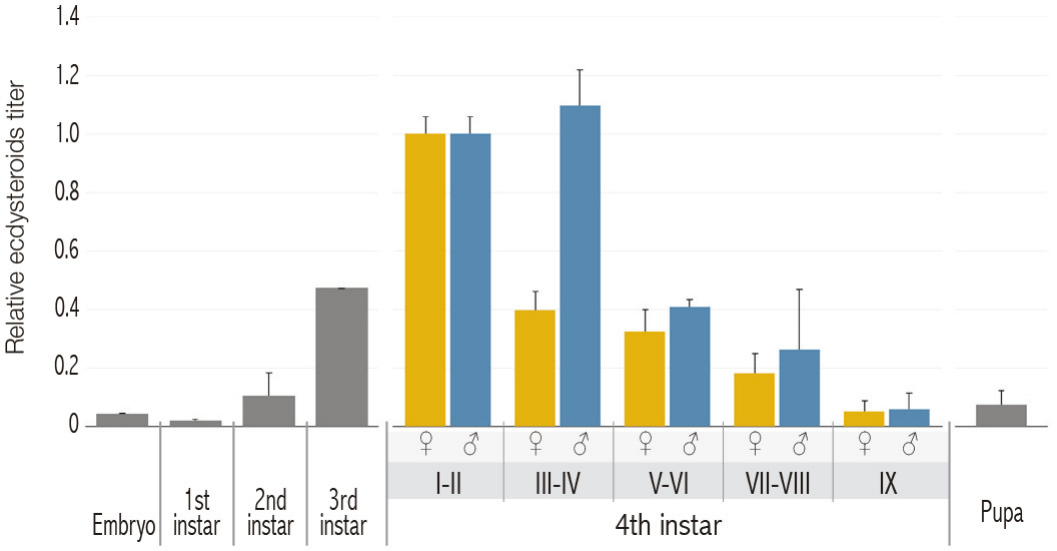
Ecdysteroid titers determination throughout *C*. *riparius* development. Ecdysteroid levels in the whole organism were analysed from embryo to pupa. Two EIA assays were performed and results are expressed as mean ± SE. A total of six egg masses and eight individuals of each developmental category were used in this study. Data corresponding the 4^th^ instar larvae are shown separately by sex. Results are shown in 20E equivalents (relative levels; pg/larva).

Ecdysteroid titers (mainly ecdysone and 20E) have been quantified by several investigators in different model insects, such as *D*. *melanogaster* or *M*. *sexta* [1,42–46]. In contrast, little is known about ecdysteroid variations in *C*. *riparius*. As a first approach, levels of total body ecdysteroids were measured using an antiserum with a maximum and similar affinity for 20E and ecdysone, and the results were expressed in 20E equivalents (Fig 5). However, according to the antiserum specifications, it also presented a high cross-reactivity percentage with 2-deoxy-20-hydroxy-ecdysone and 2-deoxy-ecdysone (88% and 63%, respectively), given that both molecules are precursors in the hormone pathway (just before ecdysone synthesis).

In contrast to what was expected according to previous studies, in which 20E titers reach their maximum at the end of the last larval stage, the highest levels of ecdysteroids in our samples were measured at the beginning of the 4^th^ instar, just after the third to fourth instar molt. High percentages of cross-reactivity could be responsible for a high level of ecdysteroids immediately after larvae molting, where the EIA kit would be detecting not only 20E but also ecdysone and its precursors. To avoid that antiserum unspecificity and seeking to clarify our ELISA results, we tried to carry out a specific determination of 20E by chromatography. However, none of the methodological approaches (different columns, different number of larvae in samples, etc.) to the analysis by HPLC / MS / MS gave a positive result, since 20E levels were below the detection limit in all cases, according to the Chromatography Service (SIDI, Universidad Autónoma de Madrid). Thus, unfortunately, the data were inconclusive and further experiments will be necessary to determine the appropriate conditions for the extraction and analysis of 20E in *C*. *riparius* samples.

## Discussion

Although the ecdysone mode of action has been well described at the molecular level in *Drosophila* and other insects [1,2,47] there is a lack of information in another model insect such as *C*. *riparius*, a widely used test organism for ecotoxicology studies [4,5]. Therefore, a major concern in developmental studies in *C*. *riparius* is to know whether ecdysone-inducible genes change their expression patterns in a stage-dependent manner or not, given that they are commonly used as molecular biomarkers [15,27,35–37,48–51].

In this work, a deep study of the ontogenetic and sex-dependent variation of such genes was carried out in *Chironomus riparius* laboratory specimens, complemented with the study of late development in natural populations.

Results obtained in this work describe for the first time a stage-dependent variation in all the ecdysone-responsive genes analyzed throughout the *C*. *riparius* development, with high inductions in the late 4^th^ instar phases, pupae and, for some genes, also in the embryo stage. The induction of both *EcR* and *usp* genes correlates well with a high activity in the embryo phase, but also with the endogenous 20E peak at the end of development, previously described in other holometabolous insects ([52] and references therein). Although data obtained in our ELISA contrast with the highest levels described in other insects for 20E at the end of larval development, our results are consistent with studies where differences in the profile of both ecdysone and 20E are demonstrated. For example, it has been described that *Gryllus bimaculatus* reaches the highest levels of ecdysone in haemolymph in earlier phases of the last larval stage, while the 20E curve shows a different trend, with the maximum peaks in males and females displaced to the late phases [53].

**Fig 6:**
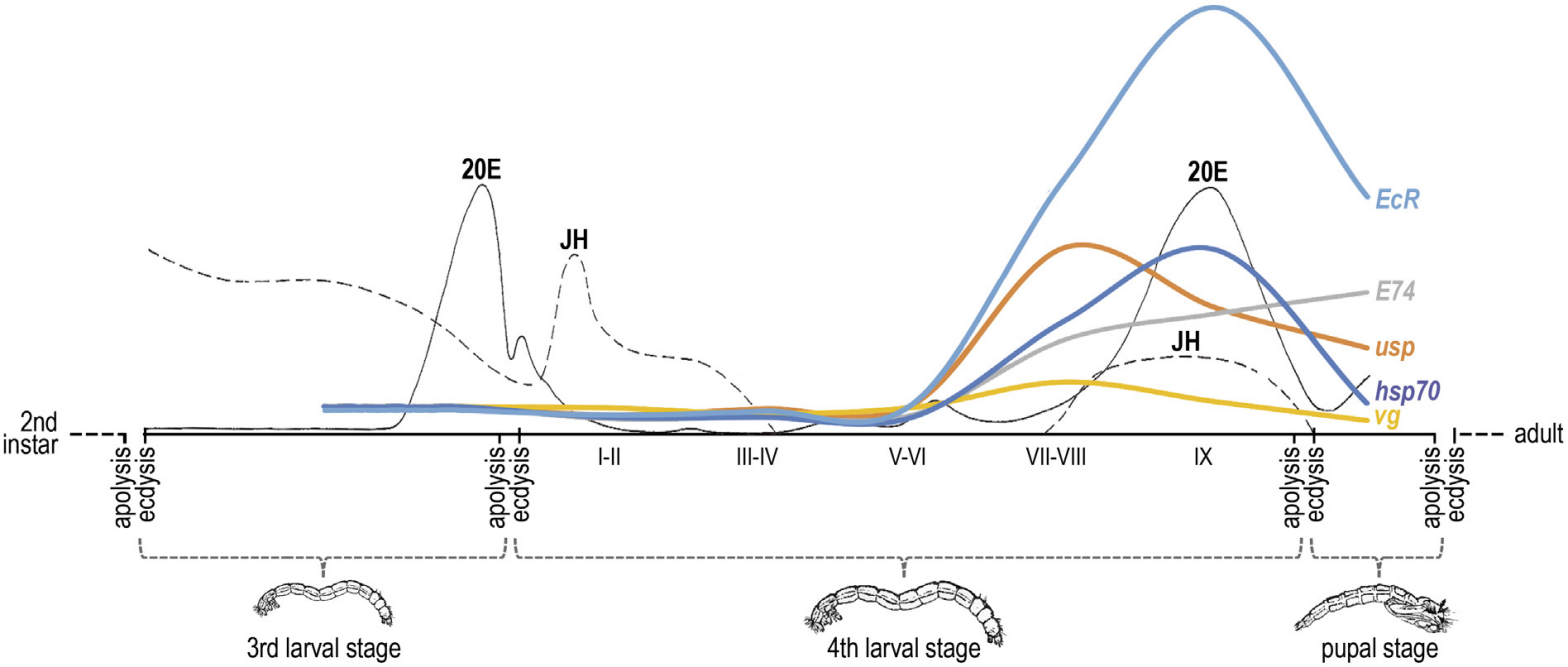
Profiles for *EcR*, *usp*, *E74*, *vg* and *hsp70* in late developmental stages in *C*. *riparius*. Schematic drawing of the life cycle of a non-biting midge (Diptera: Chironomidae, *Chironomus*) based on the results obtained in this work. Larval instars were identified by measuring head capsule widths. Age and sex of fourth instar larvae were established from the development of genital and thoracic imaginal discs according to [33]. Lines corresponding to hormone titers (20E and JH) are based on previous works [1,43–45].

In response to this hormone titer more transcripts are expected to produce the proteins that finally form the heterodimer, which acts as an ecdysone receptor. Consequently, the coordinated response measured at the end of the 4^th^ larval stage for the other analyzed genes suggests that it is in this phase where the 20E levels might increase to trigger the mechanisms that lead to metamorphosis (Fig 6).

Our results are in consonance with previous studies in *Drosophila* that describe changes in both *EcR* and *usp* genes at the pre-metamorphic stage. These studies show that *usp* fluctuations are lighter than those of *EcR*, suggesting that *EcR* is the main responsible for the response to 20E while *usp* transcribes in a constitutive manner [9,54]. Consistent with that, our results indicate a coordinated expression of both genes at the end of the 4^th^ instar with a clear higher induction of *EcR* when compared to *usp*. In addition, the transcriptional activity observed in *usp* at early 4^th^ instar larvae may be due to its constitutive expression.

*EcR* and *usp* genes have been characterized in *A*. *aegypti*, showing stage and tissue-specific responses. Whereas *usp* mRNA titer remains constant in fat body during development, both *usp* and *EcR* transcript levels fluctuate in ovaries during vitellogenesis. The kinetics of ovarian *usp* mRNA coincides with that of the ecdysteroid receptor, with high levels during the previtellogenic period and shortly after the onset of vitellogenesis [55,56]. In contrast, USP levels in the fat body remain relatively constant throughout most of the vitellogenic cycle [57,58]. The ecdysone receptor and the ultraspiracle proteins have also been described in *C*. *tentans*, where a tissue-specific regulation of the *usp* gene also occurs [59]. Data obtained in our study from total male and female larvae mRNAs support the idea that specific physiological processes in females, such as vitellogenesis, could be the reason for the different transcriptional activities of *EcR*, *usp*, and *vg*, among others, when compared to males. Interestingly, this sex and tissue-dependent fluctuations are high enough to be detected when the whole sample (including both males and females) is analyzed.

The expression pattern reported in this work suggests that the *E74* gene has a similar role at the onset of the metamorphosis in *C*. *riparius* as it does in other insects such as *D*. *melanogaster* or *M*. *sexta*. Measured levels of *E74* mRNA correlate well with timing and induction parameters necessary to reach pupation. At the time of pupal commitment, *E74* induction in *C*. *riparius* is similar to that observed at the mid-3^rd^ and the mid-5^th^ instar transitions in *D*. *melanogaster* and *M*. *sexta*, respectively, representing a classical early gene response to 20E. In *M*. *sexta*, *E74B* mRNA is detected in late larvae, when cells are becoming committed to their pupal fate [18]. In culture tissues of *M*. *sexta*, induction occurs as a direct response to a 20E peak in absence of JH, and it has been described that the exogenous addition of 20E induces a more robust response, indicating that 20E acts directly on the *MsE74* gene. A similar conclusion was deduced in *D*. *melanogaster*, where *E74A* and *E74B* mRNAs were detected during the final larval instar at the peak of ecdysteroids needed for pupation, induced *in vitro* by high concentrations of 20E [60]. Our work suggests a role of *CrE74* gene in the process of pupation, although genomic information in *C*. *riparius* is still scarce and further studies are needed to establish if different isoforms of this gene are present in this species and, in that case, the role that each one plays in the development and metamorphosis.

*Vg* cDNAs have been isolated from several insect species ([61] and references therein). The synthesis of Vg is regulated at transcriptional level and the JH and 20E hormones, and also nutrients or insulin-like peptides, have been described as yolk protein precursor regulators [62]. For example, transition from previtellogenesis to vitellogenesis corresponds to a late response of 20E in some insects, such as *D*. *melanogaster* and *Bombyx mori* [63]. *Vg* transcription is also triggered by ecdysteroids in *A*. *aegypti* [56]. However, a recent work in *Blatella germanica* has shown that JH also operates in vitellogenin expression through a cascade of genes similar to that described in the ecdysteroid hierarchy [64]. Recent studies in *T*. *castaneum* have shown that both JH and 20E are required for *vg* gene expression and, furthermore, JH regulates Vg synthesis in the fat body and 20E influences Vg synthesis through its action on oocyte maturation [65,66]. Our data support the idea that ecdysteroids induce vitellogenin expression in *C*. *riparius* 4^th^ instar larvae, suggesting that most of the vitellogenesis process takes place in this larval instar.

Although expression of *vg* gene occurs throughout all the phases described at the 4^th^ instar, the induction observed during the final stretch (VII-VIII and prepupa phases) seems to be part of the molecular hormone-related response to metamorphosis, due to the endogenous increase of 20E and the down-regulation of JH as a start signal for pupation. Since *C*. *riparius* is a non-biting species and adults do not feed, this seems to be the development stage in which vitellogenin production is needed in order to ensure adequate levels of energy reserves in the next generation of oocytes and embryos.

Wülter & Götz [33] described the formation of oocytes in *C*. *riparius*, which occurs during the 4^th^ instar of the larval stage, and gave morphological details of these changes. However, no information is available about the molecular changes that lead to oocytes production and the synthesis of energy reserves in eggs. This work constitutes the first molecular approach to vitellogenesis in *C*. *riparius*, and describes variations in the expression of a gene directly involved in the formation of the egg yolk. Our results report for the first time developmental stage and sex-related changes in the vitellogenin gene expression throughout the 4^th^ larval instar in *C*. *riparius*, which correlate in time with morphological changes described decades ago in the development of the vitello during the oocyte formation [33] and also with the sex-dependent glycogen reserves variations previously described in this organism [67]. Although further studies are needed to understand the mechanisms that underlie vitellogenesis in *C*. *riparius*, these data suggest that the final overexpression of *vg* gene could be necessary in the synthesis of egg vitellogenin in females before pupation.

Besides the physiological relevance of molecular variations of ecdysone-responsive genes related to the pupation and metamorphosis processes, it is worth pointing out that *C*. *riparius* is considered a relevant model organism for ecotoxicological assessments and therefore studies focusing on the variation of genes used as molecular biomarkers of toxicity throughout the development are particularly important. For example, different xenobiotics analogues to 20E seem to mimic the natural hormone actions, modulating the activity of the ecdysone nuclear receptor and affecting different regulatory genes directly connected with the cascade of genetic signals switched on by the ecdysone hormone (*E74* and *vg* among others) [15,27,35–37,48–51], which brings significant implications in different developmental stages in *C*. *riparius*. Regarding this, the knowledge of a stage-dependent regulation of *EcR* and the ecdysone-responsive genes in *C*. *riparius* may contribute to a more precise evaluation of the disrupting effects of EDCs in this organism.

Heat-shock proteins interact with multiple key components of signaling pathways that regulate growth and development. Certain HSPs function as chaperones by mediating intramolecular or intermolecular interactions, such as folding events or intracellular signaling and protein degradation/refolding, respectively [68,69]. This work reports for the first time in *C*. *riparius* a concomitant stage-dependent induction of the *hsp70* and *hsc70* genes and the gene encoding the ecdysone receptor, suggesting that this up-regulation is part of the early gene response to ecdysteroids due to their role in the folding and maturation of steroid hormone receptors [23]. Moreover, sex-dependent changes in the expression of both heat-shock genes correlate with the sex-dependent activity of *EcR*. As in many other organisms, the functions of HSPs in *C*. *riparius* are typically associated with stress response and tolerance [24–28], while their role during development remains unclear. Our data suggest the involvement of both genes in metabolic processes not associated with insect cellular stress, such as the ecdysone-mediated pathway that controls larval development and insect metamorphosis.

Some recent studies have shown that differential expression profiles of some ribosomal proteins take place during *C*. *riparius* life cycle, with a higher activity of these genes in embryos and pupae when compared to larvae [31,32,39]. However, these studies normally show a global expression level at larval stage, without distinguishing among the different larval instars or the phases included within the fourth instar. In this study we report for the first time a differential expression profile of *L13* gene during the 4^th^ larval instar, with a strong induction from late phases of this stage to pupa. The detection of *L13* in every developmental category studied for this research, which was expected given its role in housekeeping functions, as well as the concomitant induction of this gene together with the other of genes analyzed, are in concordance with a previous study in *D*. *melanogaster* that describes the existence of stages with high transcription and translation rates [70]. Considering the significant increase measured at the end of the juvenile stage in all the genes studied, high levels of *L13* transcript at that time could be related to the need for increased translational activity and, consequently, ribosome synthesis.

The fact that genes involved directly or indirectly in the ecdysone cascade are used as potential endpoints in the evaluation of EDCs effects reinforces the need for a more in-depth knowledge of their expression patterns during larval development in this species. Knowing the ontogenetic variations that take place in this model organism commonly used in ecotoxicity studies, it will be possible to design more accurate toxicity tests that minimize natural variations in the observed responses of such biomarkers and assess more thoroughly the effect of EDCs. These tests could be based on the use of early 4^th^ instar larva or even 3^rd^ instar larva, when intrinsic variations of such genes are scarce.

## Conclusions (and Significance)

This is the first study in *C*. *riparius* about stage-dependent variations of genes related to ecdysone response from embryo to pupa, specially during the different phases of the fourth instar larval stage. Our analyses reveal the point where *EcR* and *usp* are significantly upregulated into the 4^th^ instar. Furthermore, we have detected a coordinated overexpression of both genes with the remaining analyzed genes, suggesting that this is the moment when larvae undergo metamorphosis. This work also reveals a sex-dependent transcriptional activity of the analyzed genes during late development, some of which are associated with important physiological processes such as vitellogenesis. However, further studies are needed to establish the specific role that each gene plays in the development and metamorphosis of *C*. *riparius* males and females.

Given that *C*. *riparius* is considered a model species in ecotoxicology studies and is widely used to evaluate the impact of contaminants at the molecular level, specially the 4^th^ larval instar, our results should be taken into account in ecotoxicity testing. Indeed, most of the genes used in this work have been proposed as sensitive biomarkers of environmental stress. Thus, understanding the molecular basis of metamorphosis as well as the stage and sex-specific regulation of these ecdysone-related genes will allow us to construct more accurate models for the prediction of toxic effects, assessing more precisely what effects are due to a toxin and which are conditioned by natural physiological processes and also contributing to a more in-depth evaluation of the disrupting effects of EDCs in this organism.

## Supporting Information

S1 Fig Ontogenetic and sex-dependent variations in the expression pattern of the genes analyzed in natural population larvae and pupae. Transcriptional levels of genes, from natural population of *C*. *riparius*, involved in the ecdysone-related pathway (*EcR*, *usp*, *E74* and *vg*), the folding and maturation of steroid hormone receptors (*hsp70* and *hsc70)*, and the synthesis of the ribosomal protein L13. Gene expression was measured during 3^rd^, 4^th^ and pupa stages of development. The mRNA values were calculated relative to *actin*, *GAPDH* and *26s* as reference genes. Each bar is the mean ± SE obtained from four independent samples, each with three experimental replicates (a total of 20 larvae of each stage or phase, and separate sex were used). Significant differences among groups: *p≤ 0.05; **p≤ 0.005. Different letters indicate significant differences across groups (p≤ 0.05; p≤ 0.005).
(TIF)

S2 Fig Hierarchical clustering dendrogram of the groups established for the expression analysis in natural population larvae and pupae. Cluster analysis arranges biological samples into groups based on the expression levels. Relationships among samples are represented by a dendrogram whose branch lengths reflect the degree of similarity between them as assessed by a pairwise similarity genes response. Two groups of developmental stages or phases are clearly separated, corresponding to 3^rd^ and early 4^th^ instar larva (groups 1–4), and to late 4^th^ instar larva and pupa, respectively (groups 5–7). The root represents the whole data set. A leaf represents a single object in the data set. An internal node represents the union of all objects in its sub-tree. The weight of an internal node represents the distance between its two child nodes.
(TIF)
