# Supplementary figures and images for: Ecdysone-Related Biomarkers of Toxicity in the Model Organism Chironomus riparius: Stage and Sex-Dependent Variations in Gene Expression Profiles
Source: PLoS One. 2015 Oct 8;10(10):e0140239. doi: 10.1371/journal.pone.0140239 (PMC4598127; doi:10.1371/journal.pone.0140239)

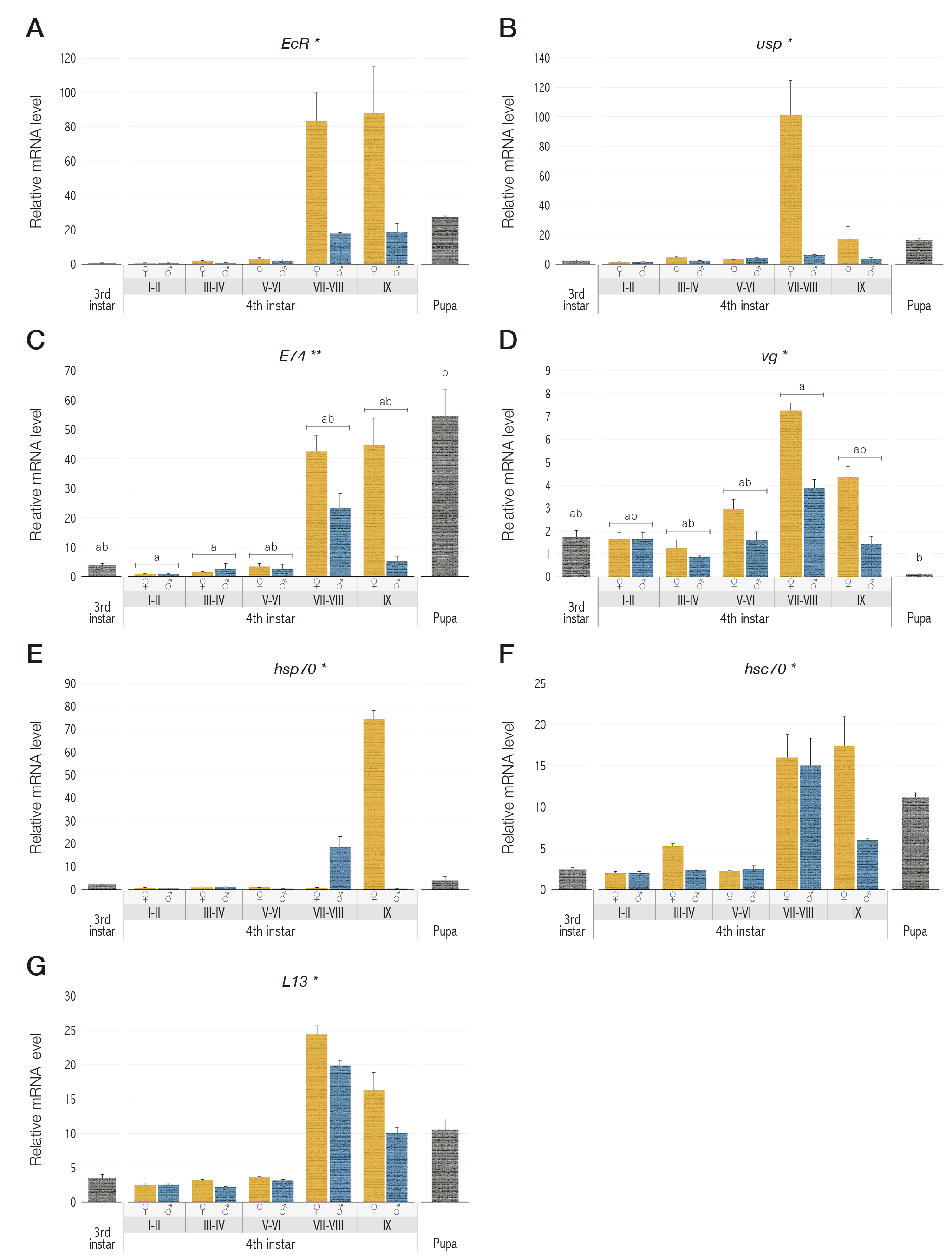

Supplement: S1 Fig — Transcriptional levels of genes, from natural population of C. riparius, involved in the ecdysone-related pathway (EcR, usp, E74 and vg), the folding and maturation of steroid hormone receptors (hsp70 and hsc70), and the synthesis of the ribosomal protein L13. Gene expression was measured during 3rd, 4th and pupa stages of development. The mRNA values were calculated relative to actin, GAPDH and 26s as reference genes. Each bar is the mean ± SE obtained from four independent samples, each with three experimental replicates (a total of 20 larvae of each stage or phase, and separate sex were used). Significant differences among groups: *p≤ 0.05; **p≤ 0.005. Different letters indicate significant differences across groups (p≤ 0.05; p≤ 0.005). (TIF) [file pone.0140239.s001.tif]

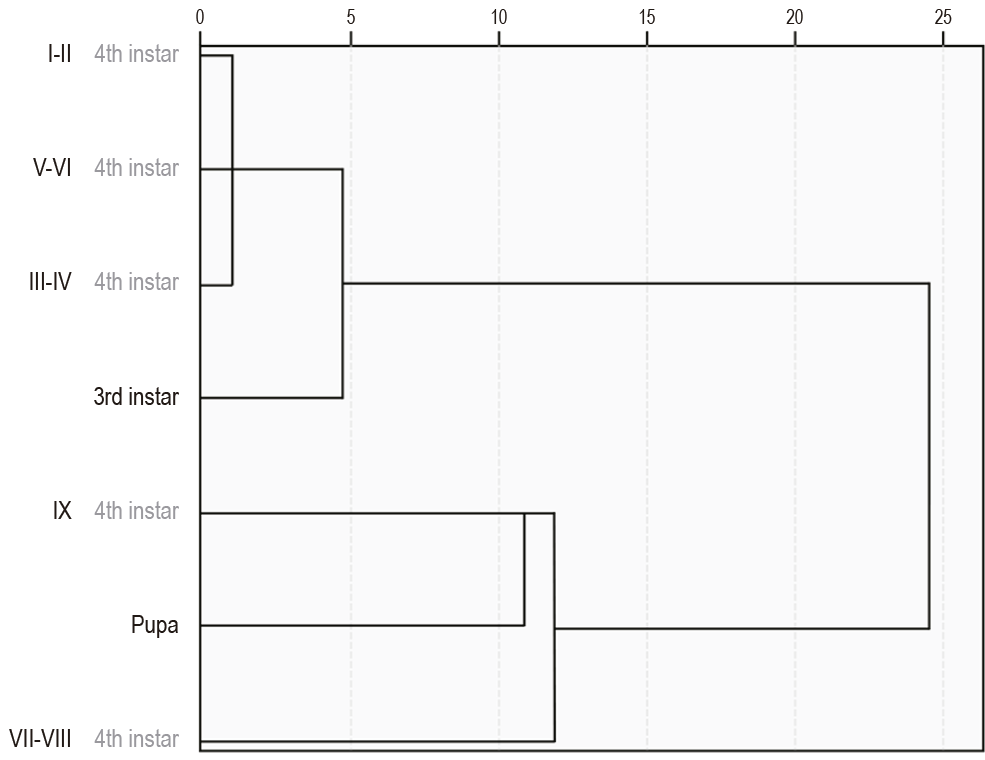

Supplement: S2 Fig — Cluster analysis arranges biological samples into groups based on the expression levels. Relationships among samples are represented by a dendrogram whose branch lengths reflect the degree of similarity between them as assessed by a pairwise similarity genes response. Two groups of developmental stages or phases are clearly separated, corresponding to 3rd and early 4th instar larva (groups 1–4), and to late 4th instar larva and pupa, respectively (groups 5–7). The root represents the whole data set. A leaf represents a single object in the data set. An internal node represents the union of all objects in its sub-tree. The weight of an internal node represents the distance between its two child nodes. (TIF) [file pone.0140239.s002.tif]
